# Endoplasmic reticulum in health and disease: the 12th International Calreticulin Workshop, Delphi, Greece

**DOI:** 10.1111/jcmm.13413

**Published:** 2017-11-21

**Authors:** Aristidis S. Charonis, Marek Michalak, Jody Groenendyk, Luis B. Agellon

**Affiliations:** ^1^ Biomedical Research Foundation of the Academy of Athens Athens Greece; ^2^ Department of Biochemistry University of Alberta Edmonton Alberta Canada; ^3^ School of Human Nutrition McGill University Ste. Anne de Bellevue Quebec Canada

**Keywords:** calreticulin, calnexin, endoplasmic reticulum, calcium, unfolded protein response (UPR), diseases

## Abstract

Starting from 1994, every 2 years, an international workshop is organized focused on calreticulin and other endoplasmic reticulum chaperones. In 2017, the workshop took place at Delphi Greece. Participants from North and South America, Europe, Asia and Australia presented their recent data and discussed them extensively with their colleagues. Presentations dealt with structural aspects of calreticulin and calnexin, the role of Ca^2+^ in cellular signalling and in autophagy, the endoplasmic reticulum stress and the unfolded protein response, the role of calreticulin in immune responses. Several presentations focused on the role of calreticulin and other ER chaperones in a variety of disease states, including haemophilia, obesity, diabetes, Sjogren's syndrome, Chagas diseases, multiple sclerosis, amyotrophic lateral sclerosis, neurological malignancies (especially glioblastoma), haematological malignancies (especially essential thrombocythemia and myelofibrosis), lung adenocarcinoma, renal pathology with emphasis in fibrosis and drug toxicity. In addition, the role of calreticulin and calnexin in growth and wound healing was discussed, as well as the possible use of extracellular calreticulin as a marker for certain diseases. It was agreed that the 13th International Calreticulin Workshop will be organized in 2019 in Montreal, Quebec, Canada.

**Table:** 

• Introduction
• Calreticulin and calnexin: structural aspects
• Endoplasmic reticulum: Ca^2+^ signalling and autophagy
• Endoplasmic reticulum stress and unfolded protein responses (UPR)
• Immune system
• Endoplasmic reticulum, molecular chaperones and human diseases
• Short presentations by young researchers
Conclusions

## Introduction

The International Calreticulin Workshop is named after the endoplasmic reticulum (ER) chaperone known as calreticulin (Calr), first described in 1974 as a high‐affinity Ca^2+^ binding protein in the muscle sarcoplasmic reticulum 1. Motivated by a passion for the protein and the belief that it is a fundamental component of the ER, 20 years later in 1994, Marek Michalak organized in Banff, Canada, the 1st International Calreticulin Workshop. From that point on, the meeting has taken place biannually with workshops held in Italy, Switzerland, USA (twice), UK, Chile, Canada (4 times) and Denmark. Since 1994, the Workshop evolved into an international forum to highlight many aspects of the structure and function of the ER and the protein/lipid quality control machinery. After 23 years of the Calreticulin Workshop tradition, the 12th meeting was held in Delphi, Greece, in 18–22 May 2017 hosted by Aris Charonis (Athens, Greece). In Delphi, major advances were discussed in the area of quality control of the protein secretory pathway, ER chaperones, ER Ca^2+^ signaling, unfolded protein responses (ER stress coping response), proteostasis and lipidostasis, Calr and calnexin (Canx) in health and disease, including an emphasis on myeloproliferative neoplasms (MPN), cardiovascular health, neurodegenerative diseases, cancer, wound healing, immunity, the pathology of protein folding diseases, congenital diseases and metabolic disorders.

Many vital cellular processes occur in the ER, notably Ca^2+^ storage and release, lipid synthesis, and protein synthesis, folding and post‐translational modification 2, 3, 4. The ER is also the first compartment in the protein secretory pathway involved in cellular signalling and organelle–organelle communication including activation of gene transcription. The ER is intimately involved in Ca^2+^ signalling and communication with the plasma membrane Ca^2+^ channels. Importantly, the ER is a multifunctional organelle able to detect and integrate incoming signals, modulate its own luminal dynamics and generate output signals in response to environmental changes. Disruption of ER homoeostasis (proteostasis and lipidostasis) therefore results not only in cellular disease but also has detrimental effects at organ and systemic levels as well. To support its diverse cellular functions, the ER contains many luminal and integral membrane proteins that have dynamic interactions with membrane lipids as well as lipid metabolites. Calr is a major Ca^2+^ buffering chaperone and ER‐resident protein involved in a variety of biological processes including quality control of protein folding, regulation of Ca^2+^ homoeostasis and MHC class I antigen processing. Calr plays an important role in both the innate and adaptive immune response. Cell surface Calr impacts the phagocytosis of apoptotic cells and has potential application as a therapeutic for diabetic wound healing and cancer therapy. The ER membrane protein Canx, which has a high degree of structural similarity to Calr, works in conjunction with Calr as molecular chaperones in the ER protein quality control system. Several aspects of Calr and Canx structure and function, and their impact on ER homoeostasis, were discussed at the Delphi meeting.

## Calreticulin and calnexin: structural aspects

Using chemical cross‐linking combined with mass spectrometry (XLMS), the group of Peter Hojrup (Odense, Denmark) has probed the structure of the flexible arms of Calr and the influence of Ca^2+^. Under cellular stress conditions, Calr easily multimerizes, and through XLMS, a model of the protein dimer was determined. The dimer forms in parallel fashion, and the P‐domain of Calr is involved in intermolecular bonds. This model fits with the presence of higher orders of multimerization observed and supports the ability of Calr to interact with many partners. While most of these interactions are short‐lived, and thus difficult to detect, Peter's group has been able to obtain preliminary interaction data for a number of proteins, including protein disulphide isomerase 3 (PDIA3) and complement component 1 (C1q).

Using NMR spectroscopy, Kalle Gehring's group (Montreal, Canada) presented elegant studies on direct binding of oxidoreductase ERp29 (PDIA9) to the P‐domain of Canx and the role of D348 in Canx, which when mutated abrogated interactions between Canx and PDIA9, PDIA3 and cyclophilin B (CypB). Using X‐ray crystallography, the structures of the D‐domain of PDIA9 bound the P‐domains of Calr and calmegin were presented. These structures reveal that the tip of the P‐domain functions as a plurivalent adapter to bind different folding factors. The structural diversity of the bound chaperones suggests that they became specialized for glycoprotein folding through convergent evolution of their P‐domain binding sites. In addition, this group has used negative‐stain electron microscopy to determine the structure at 18 Å resolution of UDP‐glucose:glycoprotein (UGGT) from *Drosophila melanogaster*. Three‐dimensional reconstruction revealed a cage‐like structure with a large central cavity and flexibility that precluded determination of a high‐resolution structure. It was suggested that the central cavity contains the catalytic site and hydrophobic surfaces to enhance the binding of misfolded substrates and exclude hydrophilic, folded proteins, possibly the mechanism behind UGGT function 5.

Gisou van der Goot's group (Lausanne, Switzerland) discussed the role of palmitoylation in the ER. The ER hosts many Asp‐His‐His‐Cys (DHHC) domain‐containing palmitoyltransferase enzymes that mediate protein palmitoylation. Palmitoylation occurs on cysteine residues and allows soluble proteins to become peripheral membrane proteins. DHHC6 is of particular interest as it modifies key ER proteins involved in protein folding and quality control, Ca^2+^ homoeostasis and ER architecture. Data were presented on the regulation of DHHC6 function, some of its targets and consequences of palmitoylation. Approximately 10% of the human proteome undergoes palmitoylation and targets include major channels and receptors, enzymes and signalling molecules 6, 7, 8.

## Endoplasmic reticulum: Ca^2+^ signallingand autophagy

Nicolas Demaurex (Geneva, Switzerland) focused on the ER Ca^2+^‐sensing protein stromal interacting molecule 1 (STIM1), acting as an intracellular ligand to control the gating of Ca^2+^‐permeable channels of the Ca^2+^ release‐activated calcium modulator (ORAI) and transient receptor potential cation channel (TRPC) families. STIM/ORAI coupling occurs at membrane contact sites, which are evolutionarily conserved structures that sustain non‐vesicular lipid transfer at sites of close apposition (~10–20 nm) between the ER and other cellular membranes. In neutrophils, STIM/ORAI interactions occur on phagocytic vacuoles and enhance the efficiency of phagocytosis by promoting actin dynamics and by enabling phagocytic vacuoles to gain oxidative and lytic properties *via* Ca^2+^‐dependent fusion with granules and lysosomes. Using ultrastructural, genetic and functional approaches, the Demaurex group showed that STIM1 increases the formation of ER‐phagosome contact sites to generate local Ca^2+^ elevations that promote the phagocytic process. At ER‐phagosome contact sites, STIM1 triggers the opening of phagosomal Ca^2+^ channels and synergizes with its adapter protein junctate to promote inositol trisphosphate (InsP_3_)‐mediated Ca^2+^ release from the recruited ER stores. In dendritic cells, *Stim1* ablation reduced local and global Ca^2+^ signals as well as cross‐presentation and chemotaxis without altering cell differentiation, maturation or phagocytic capacity. Phagosomal proteolysis and insulin‐regulated aminopeptidase (IRAP) activity, as well as fusion of phagosomes with endosomes and lysosomes were impaired by STIM1 deficiency. Local Ca^2+^ signals occurring at membrane contact sites between the ER and phagosomes may regulate the efficiency of bacterial killing by neutrophils and the ability of dendritic cells to induce antiviral and anticancer immunity.

Jan Parys (Leuven, Belgium) emphasized that Ca^2+^ handling by the ER and especially *via* the InsP_3_ receptor/Ca^2+^ release channels plays a crucial role in the autophagy process. Autophagy is a conserved, catabolic pathway that under basal conditions assures cell homoeostasis by degrading protein aggregates, long‐lived proteins and dysfunctional organelles and is up‐regulated during stress conditions. In follow‐up of previous work, Jan Parys presented the latest findings concerning the Ca^2+^ dependence of resveratrol‐induced autophagy and of autophagy triggered by the ER stress inducer azetidine carboxylic acid (AZC). Interestingly, resveratrol‐induced autophagy is even strictly dependent on the InsP_3_ receptor, as a mammalian cell line deficient in InsP_3_ receptors lost its sensitivity for resveratrol‐induced autophagy 9. The relationship between resveratrol and AZC‐induced autophagy and Ca^2+^ should be further explored, as although both compounds affect ER Ca^2+^‐store handling, they do so in a different way.

Maurizio Molinari (Bellinzona, Switzerland) reported on mechanisms operating in mammalian cells to clear aggregated polypeptides generated in the lumen of the ER. Eukaryotic cells host two major catabolic pathways, the ubiquitin proteasome system (UPS) and autophagy. The majority of misfolded proteins produced in the ER are retrotranslocated across the ER membrane and degraded by the UPS in a series of processes defined as ER‐associated degradation. It is postulated that the UPS‐resistant aggregates accumulating in the ER lumen are removed by macroautophagy. About 40 autophagy (ATG) gene products regulate formation of double membrane structures, the phagophores, that capture cytosolic material, mature to autophagosomes that eventually fuse with lysosomes to eliminate their content (damaged organelles or aggregated proteins). Molinari's group showed that generation of double membrane phagophores and autophagosomes is dispensable for clearance of misfolded ordered and proteasome‐resistant polymers generated in the ER. These are cleared from cells *via* direct ER to lysosomes transport defined as ER‐ to lysosome‐associated degradation (ERLAD).

Much remains to be understood about the Ca^2+^‐binding characteristics of ER chaperones and how Ca^2+^ binding affects substrate interactions with ER chaperones. Calr is known to have multiple low‐affinity Ca^2+^‐binding sites within an acidic C‐terminal region that is mutated in myeloproliferative neoplasms (MPN) 10, 11. In these mutants, the acidic C‐terminal region of calreticulin is altered to a basic sequence, along with a loss of the C‐terminal ER retention‐specifying KDEL sequence. It has been previously shown by Malini Raghavan (Ann Arbor, USA) that murine Calr lacking the C‐terminal acidic region has enhanced tendency to form oligomers 12. Consistent with those findings, the Raghavan laboratory reports that purified versions of the MPN mutants are less stable and elute from gel filtration columns as oligomeric/aggregated protein compared to wild‐type human Calr. Similar results were observed in transfected HEK cell lysates. These findings, of the increased propensity of the MPN mutants of Calr to self‐associate, might explain the previous studies showing that calreticulin MPN mutants can drive cytokine‐independent activation of the thrombopoietin receptor (TPOR) 13. The Raghavan laboratory also showed that MPN mutants have reduced abilities to chaperone both TPOR and major histocompatibility complex (MHC) class I molecules, as steady levels of both proteins are negatively impacted by the presence of the mutations. Overall, pathological alterations to the Ca^2+^‐binding acidic region of Calr are shown to reduce stability, induce self‐association and alter cellular chaperone function.

The ER chaperone BiP also contains acidic C‐terminal region, but the relevance of this region to Ca^2+^ binding by BiP and the formation of BiP oligomers was unknown. Using isothermal titration calorimetric studies, the Raghavan laboratory showed that BiP contains low‐affinity Ca^2+^ binding sites in the ER range of Ca^2+^ concentrations. The role of the C‐terminal acidic region in low‐affinity Ca^2+^ binding by BiP is being investigated. BiP oligomers, previously described, are destabilized by truncations of the C‐terminal acidic region of BiP. Thus, the C‐terminal regions of BiP and Calr regulate their self‐associations, but in opposite directions.

## Endoplasmic reticulum stress and unfolded protein responses (UPR)

The UPR is an essential cell signalling system that detects the presence of misfolded proteins within the ER, and then initiates a number of cellular responses that aims restore ER protein homoeostasis 14, 15. The primary step in UPR is the detection of misfolded proteins, the precise mechanism of which remains unclear. Maruf Ali (Imperial College London) presented data using a reconstituted in vitro UPR system that showed a non‐canonical interaction between BiP and IRE1 occurred solely *via* the ATPase domain of BiP, which was independent of nucleotides. Then using misfolded protein substrate, CH1, they demonstrated that it bound solely to the canonical substrate‐binding domain of BiP, and not to the luminal domain of Ire1 16. When BiP and Ire1 form an interaction, the addition of CH1 causes the dissociation of the complex *via* a conformational change. This observation forms the basis of the proposed allosteric model for UPR induction that provides a rationale for how UPR detects misfolded proteins^3^.

Characterization of the IRE1α interactome followed by functional validation by the group of Claudio Hetz (Santiago, Chile) and his collaborators unveiled several novel regulators of the UPR, highlighting the ER chaperone heat‐shock protein 47 (Hsp47) as the major hit. Cellular and biochemical analyses revealed that Hsp47 instigates IRE1α signalling through a physical interaction. At the molecular level, Hsp47 directly binds to the ER luminal domain of IRE1α with high affinity, displacing the negative regulator BiP from the complex to facilitate its oligomerization. Regulation of IRE1α signaling by Hsp47 is evolutionary conserved as validated using genetic manipulation in fly and mouse models of ER stress. Therefore, it was concluded that Hsp47 adjusts IRE1α signalling by fine‐tuning the threshold to engage an adaptive UPR.

## Immune system

Robert Binder (Pittsburgh, USA) presented recent findings from his group regarding the regulation of the signalling cascades within antigen‐presenting cells, triggered by ER heat‐shock proteins (HSP) 17. More specifically, they have shown that when released from aberrant cells, these HSPs engage their receptor CD91 expressed on antigen‐presenting cells. CD91 allows for cross‐presentation of the HSP‐chaperoned peptides and activation of intracellular signalling pathways leading to costimulation 18. These HSP–peptide complexes, as a single entity, have the capacity to prime T‐cell responses with specificity for aberrant cells including tumours and infected cells. Using mouse models of cancer, they have shown how these endogenous proteins, together with CD91, influence immunosurveillance of tumours 19. Mice lacking CD91 on their dendritic cells develop tumours more frequently than wild‐type mice when exposed to carcinogens. Tumours developing in the knockout mice are less immunoedited. The HSP receptor, CD91, is polymorphic, and initial evidence was presented unravelling how each allele affects HSP binding and their correlation to development of cancer in humans, offering a novel outlook on how HSPs control the T‐cell response in the context of autoimmune disease and immunosurveillance of cancer.

C. James Lim (Vancouver, Canada) discussed how cell adhesion is a physiologically relevant stimulus shown to increase the interaction of Calr with α‐integrins *via* the GFFKR motif, a possible implication of cytosolic Calr as a mediator of drug resistance 20. Indeed, T‐lymphoblasts lacking Calr expression exhibit increased susceptibility to drug‐induced apoptosis and reduced influx of extracellular Ca^2+^. His group then assessed whether integrin function can modulate surface Calr levels in immunogenic cell death (ICD) 21. When stimulated to engage integrin substrates, T‐lymphoblasts treated with an ICD inducer exhibited decreased surface Calr compared with cells under non‐adherent conditions. The inhibitory effect on surface Calr was recapitulated for cells in suspension and treated with agents that stimulate integrin activation. Similarly, cells expressing the truncated α‐integrin with GFFKR as the cytosolic tail also exhibited low surface Calr when treated with ICD inducers under non‐adherent conditions. Using permeabilization techniques that distinguish between cytosolic and ER staining, they found that ICD inducers promoted the accumulation of cytosolic Calr with negligible change in total Calr, suggesting that integrin‐mediated inhibition of surface Calr was due to reduced cytosolic to surface translocation. T‐lymphoblasts cotreated with an ICD inducer and integrin activators exhibited reduced phagocytosis by macrophages when compared with treatment with only the ICD inducer.

Peter Cresswell (New Haven, USA) presented on protein entry into the cytosol during antigen cross‐presentation. The precise mechanism(s) for this event remain obscure, but previous work from his group argues that the property is not restricted to dendritic cells (DCs) or even to classical antigen‐presenting cells. To further enhance our understanding of the pathway, they have developed a glycosylated derivative of *Renilla* luciferase that is virtually inactive until the glycan is enzymatically removed by protein N‐glycanase (PNGase), which converts the asparagine to which the glycan is attached into an aspartic acid residue. Cytosolic entry after phagocytosis or endocytosis followed by deglycosylation by endogenous cytosolic PNGase liberates the activity of the enzyme, providing a probe to analyse the translocation function as well as downstream processes that affect the reconstitution of cytosolic activity. Characterization of the luciferase probe and its potential for unravelling the cellular processes involved in liberating its cytosolic activity were discussed.

Leslie Gold's group (New York, USA) in collaboration with Paul Eggleton's group (Exeter, UK) presented data suggesting that extracellular Calr (eCalr) specifically sequesters and binds to lipopolysaccharide (LPS), enhancing both cellular and innate immune processes. This interaction may represent a combined damage (eCalr) and pathogen (LPS)‐associated molecular pattern danger signalling molecule to the immune system. It appears that LPS binds multimeric eCalr and may even induce aggregation of this protein. Notably, eCalr dose dependently bound to immobilized LPS by ELISA, which was increased twofold by Ca^2+^.

## Endoplasmic reticulum, molecular chaperones and human diseases

Haemophilia A (HA) results from deficiency in coagulation factor VIII (FVIII). Preferred therapy requires prophylactic protein replacement with recombinant‐derived FVIII (rFVIII). Although several proteins that interact with FVIII within the ER have been identified, including the ER chaperones BiP, Canx, Calr and the ER to Golgi intermediate compartment members LMAN1/ERGIC‐53 and multiple coagulation factor deficiency 2 (MCFD2), both the mechanism and the other factors governing proper folding and efficient secretion of FVIII are still poorly understood. Randal Kaufman (La Jolla, USA) provided evidence that FVIII forms aggregates within the ER that have a structure characteristic of amyloid. Significantly, the aggregation is reversible in a process that requires cellular energy in which the aggregates can be refolded properly to secrete functional FVIII. These aggregates can also be dissociated *in vitro* by incubation with hydrolysable ATP. FVIII aggregation in the cell causes mitochondrial oxidative stress, and a mitochondrial‐targeted antioxidant reduces oxidative stress and promotes solubility and secretion of FVIII. FVIII gene transduction into hepatocytes in mice also causes FVIII aggregation, which when combined with a high‐fat diet treatment leads to hepatocellular carcinoma. The aggregation of FVIII in the ER presents a unique example of a natural protein that is prone to amyloid formation in the ER, indicating that cellular biochemical machinery exists to dissociate these aggregates for productive folding and secretion of functional protein.

Giannis Spyrou (Linköping, Sweden) presented data on ERdj5 (PDIA19), an ER chaperone protein belonging to the thioredoxin superfamily with a highly potent reductase activity and an integral part of the ER‐associated protein degradation system (ERAD), which binds to BiP to facilitate the cleavage of S‐S bonds in terminally misfolded proteins before they can be translocated to the cytosol for proteasomal degradation. Presented data revealed that ERdj5 function affects metabolic pathways, immune responses and protein aggregate formation in various afflicted tissues. The ERdj5 gene knockout mice develop an obese and diabetic phenotype with elevated blood glucose, insulin resistance, altered lipid and carbohydrate metabolism rates compared to the wild‐type controls. These mice also develop inflammatory lesions in the salivary glands resembling the pathological manifestations of Sjögren's syndrome and are positive for antinuclear autoantibodies in the serum. The presented studies provided evidence for the crucial connection between the ER protein quality control system and diverse classes of human disease, such as NAFLD, diabetes and autoimmunity.

Pius Nde (Nashville, USA) presented his latest work on *Trypanosoma cruzi*, the causative agent for Chagas diseases. *T. cruzi* trypomastigotes up‐regulate the expression of thrombospondin‐1 (TSP‐1) which is important in the process of cellular infection. They have shown that the N‐terminal domain of TSP‐1 (NTSP) interacts with *T. cruzi* Calr (TcCalr) in a complex of purified parasite membrane surface proteins, thus predicting one of the domains of TSP‐1 molecule that is important in mediating interaction with TcCalr. Using fluorescence activated cell sorting (FACS) and membrane protein expression analysis, they showed that transgenic parasites had more TcCalr on their surface compared to control parasites. Invasive trypomastigotes overexpressing TcCalr showed a higher level of infectivity compared to control parasites. Therefore, TcCalr is a virulent factor that enhances *T. cruzi* infection.

Evidence suggests that changes in expression of ER chaperones can influence cell surface protein stabilization and function in the nervous system. Marek Michalak (Edmonton, Canada) presented the latest findings on a potential role of Canx in the pathology of multiple sclerosis.

Sonam Parakh (Sydney, Australia) presented the latest findings from Julie Atkin's laboratory, regarding amyotrophic lateral sclerosis (ALS). There is increasing evidence that ER chaperones PDIA1 (PDI) and PDIA3 (ERp57) are protective against neurodegenerative diseases related to protein misfolding, including ALS, which is a rapidly progressing disorder affecting motor neurons in the brain, brainstem and spinal cord, resulting in paralysis and death usually 2–5 years post‐diagnosis. Work from the group has demonstrated that overexpression of PDI is protective against mutant superoxide dismutase (SOD1) misfolding and aggregation, ER stress and apoptosis, in neuronal cell lines. PDI family members are protective against other ALS‐associated proteins and against multiple features characteristic of cellular pathology in neuronal cells and primary neurons, including misfolded protein aggregation, mislocalization to the cytoplasm, ER stress, inhibition of ER–Golgi transport and induction of apoptosis. PDI family members are protective *in vivo* in zebrafish models of ALS. Therefore, therapeutics based on PDI may be attractive candidates for ALS. However, in addition to its protective functions, aberrant, toxic roles for PDI have recently been described. These functions need to be fully characterized before effective therapeutic strategies can be designed.

Eric Chevet (Rennes, France) talked about proteostasis imbalance as an emerging major hallmark of cancer, driving tumour aggressiveness as genetic and pharmacological evidence suggests that the ER plays a critical role in cancer development. This concept was evaluated in the case of glioblastoma multiform (GBM), the most lethal primary brain cancer with an overall survival of 15 months and no effective treatment. Previous work from the group has demonstrated that the ER stress sensor IRE1α contributes to GBM progression, impacting tissue invasion and tumour vascularization. IRE1α also regulates the stability of certain miRNAs and mRNAs through a process known as RIDD. Somatic mutations in the IRE1α gene have been identified in GBM and other forms of cancer. Data were presented regarding the contribution of IRE1α signalling to GBM based on the systematic comparison of mutant forms identified in cancer, and demonstrated its significance to the disease. A novel mutation associated with GBM was uncovered, with functional consequences to tumour formation. Taking advantage of the specific signalling outputs of the RNase domain of IRE1α engaged by distinct GBM‐related mutations, specific expression signatures were defined that were confronted to human GBM transcriptomes. This approach allowed them to demonstrate the antagonistic roles of XBP1 mRNA splicing and RIDD on tumour outcome. These data led to the demonstration of a dual role of IRE1α downstream signalling in cancer and open a new therapeutic window to abrogate tumour progression 22.

Alexandre Theocharides (Zurich, Switzerland) presented data on Philadelphia‐negative MPN, chronic haematopoietic stem cell disorders characterized by increased proliferation of one or more haematopoietic cell lineages. MPN are characterized by frequent somatic mutations in the Janus kinase 2 (JAK2) and *Calr*, functionally a chaperone involved in the correct folding of N‐linked glycoproteins 10, 11. The presented data showed that MPN patients with homozygous *Calr* mutations develop a maturation defect in myeloperoxidase (MPO), a glycoprotein folded by *Calr* 23. Acquired MPO deficiency in MPNs is a rare phenomenon first described four decades ago 24, and the studies of the group provide the molecular correlate associated with this phenomenon. These data provide evidence that *Calr* mutations can affect the chaperone function of Calr. The mechanism(s) of MPO deficiency in MPN and the possible implications for future basic and clinical research in the field were discussed.

Ilyas Chachoua (Brussels, Belgium) and Anita Roy (Brussels, Belgium) from Stefan Constantinescu laboratory presented data on how mutants of Calr are associated with MPNs, essential thrombocythemia and myelofibrosis. The phenotype induced by Calr mutants is due to pathologic activation of the thrombopoietin receptor (Mpl, TpoR) in the secretory pathway and at the cell surface. Localization of mutant Calr in ERGIC, Golgi and surface was demonstrated by confocal immunofluorescence microscopy. Using FRAP (Fluorescence Recovery after Photobleaching), a change in mobility of TpoR upon expression of Calr mutants at the plasma membrane was shown. Using structure‐guided mutagenesis as well as serial truncations from the C‐terminus of Calr mutants, the sequences required by TpoR and Calr mutants for interaction and eventual activation of TpoR‐JAK‐signal transducer and activator of transcription 5 (STAT5) signalling were defined. Using CRISPR/Cas9 technology, knockin heterozygous cell line and mouse models of murine Calr del52 and del61 were generated. Although the frameshift in the mouse Calr leads to a slightly different sequence, the biophysical features remain similar to those of human Calr mutants and effects on TpoR appear similar. Early data indicate the appearance of a thrombocytosis phenotype and histological features reminiscent of MPNs in the mouse models. These data further support the notion that the major driver event induced by mutant Calr is activation of TpoR 13, 25, 26, 27.

Activation of ER stress coping responses has been associated with nephrotoxicity and kidney fibrosis. Cyclosporine (CsA) is an immunosuppressant widely used in organ transplantation and in treatment of various autoimmune diseases. CsA binds to cyclophilin A and the complex associates with and inhibits calcineurin. CsA‐dependent inhibition prevents activation of promoters of T cell activation and overall immune response. Prolonged intake of CsA induces secondary side effects including fibrosis and chronic nephrotoxicity. The group of Jody Groenendyk and Marek Michalak (Edmonton, Canada) discovered that CsA interacts with prostaglandin endoperoxidase and cyclooxygenase 2 (COX‐2), an inducible, ER‐associated enzyme. The CsA‐COX‐2 complex binds to IRE1α to enhance its activity and contributes, at least in part, to development of fibrosis. Increased COX‐2 activity is associated with renal tissue damage and poor outcome for kidney transplant patients. COX‐2 enzyme is also up‐regulated during cardiac allograft rejection and correlates with a poor outcome. CsA treatment causes an increase in tumour necrosis factor‐α (TNFα) secretion, induction of the fibrotic cascade and a disruption in the redox balance. Tauroursodeoxycholic acid (TUDCA) is a bile acid and proteostasis promoter that is shown to reduce the UPR and prevent cardiac fibrosis. Data were presented showing that application of TUDCA prevents fibrosis in a nephrotoxic mouse model system.

Transforming growth factor‐β (TGF‐β) is a key factor in the pathogenesis of diabetic nephropathy, a leading cause of end‐stage renal disease. Joanne Murphy‐Ullrich's group (Birmingham, USA) previously showed that Calr is important for collagen transcription, secretion and assembly into the extracellular matrix and that Calr is critical for TGF‐β stimulation of type I collagen transcription through Calr's action on ER calcium release and NF‐AT activation. In recent work, her group used Calr fl/fl mice to knockdown Calr expression in a tissue‐specific manner in adult mice through targeted ultrasound delivery of cre‐recombinase plasmid and showed that Calr knockdown reduced neointimal hyperplasia and collagen in response to acute vascular injury in the carotid artery 28, 29, 30. She presented data showing that knockdown of Calr expression in the kidney of uninephrectomized Calr floxed mice made diabetic with streptozotocin‐reduced renal dysfunction as measured by the urinary albumin/creatinine ratio and glomerular and tubulointerstitial fibrosis. The presented data identify Calr as an important regulator of TGF‐β‐stimulated extracellular matrix production in the diabetic kidney.

Previous work in Aris Charonis group (Athens, Greece) has established an increase in Calr expression in a mouse model of fibrosis localized exclusively in tubular epithelial cells. In a human tubular epithelial cell line, proteomic analysis was performed comparing control and overexpressing Calr cells. Among several alterations in expression, the up‐regulated family of 14‐3‐3 proteins was selected for further study. These proteins are considered docking proteins, mediators of several signalling pathways and their roles in nephropathies remain largely unknown. Proteomic analysis data were confirmed by PCR and Western blotting. In addition, cell lines where Calr expression was down‐regulated by sh‐RNA exhibited in PCR and Western blotting down‐regulation of all members of the 14‐3‐3 family. These *in vitro* studies were confirmed *in vivo*, in the animal fibrotic model biochemically and morphologically. Among them, the σ‐isoform exhibited the highest expression. In addition, transgenic animals that were heterozygous for Calr were expressing reduced amounts of 14‐3‐3 proteins. Furthermore, studies using human biopsy material from patients suffering from IgA nephropathy, membranous nephropathy, lupus nephropathy and antineutrophil cytoplasm‐associated (ANCA) nephropathy revealed a pronounced up‐regulation of the expression of 14‐3‐3 proteins compared to control biopsies, almost exclusively in distal tubule epithelial cells. Further studies were proposed to establish the 14‐3‐3 family as an important diagnostic marker.

Chronic wound healing, which occurs as a consequence of diabetes, in pressure ulcers, and venous stasis ulcers, is characterized by a paucity of granulation tissue due to the lack of recruitment and proliferation of cells necessary for healing. Leslie Gold's group (New York) has shown 31, 32 that topical application of the intracellular ER chaperone, Calr, enhances the rate and quality of wound healing in animal models and that *in vitro* exogenous Calr (eCalr) can ameliorate most cellular defects that impair the healing of diabetic wounds. eCalr promotes cellular migration and proliferation of keratinocytes to resurface the wound, recruits macrophages to phagocytose cellular debris, attracts fibroblasts into the wound and induces extracellular matrix (ECM) production to reconstruct the wound defect. It was shown that eCRT could not induce ECM proteins or migration of Calr null MEFs. A novel paradigm evolved showing that intracellular ER Calr (iCalr) and eCalr are both required for the induction of collagen, fibronectin and elastin synthesis for tissue regeneration in the following manner: eCalr binds to the surface receptor LRP1 (CD91), LRP1 signalling induces TGF‐β1 and TGF‐β3 proteins that are released from the cell and subsequently in an autocrine manner, induce canonical TGF‐β signalling *via* Smad 2 and 3 transcription factors for iCalr‐dependent ECM induction. The data presented showed the importance of the convergence of intracellular and extracellular Calr in ECM induction essential for wound healing and tissue regeneration.

Following excisional wound repair, epidermal appendages, such as hair follicles and sebaceous glands are not recovered in these deep tissue wounds. In a separate presentation, Leslie Gold (New York) reported that topical application of Calr to experimental wounds in diabetic mice induced melanin‐containing hair follicle neogenesis, whereas buffer‐treated wounds showed normal scar tissue formation. This finding implies that Calr promotes a regenerative healing process rather than classic adult wound repair. *In vitro* studies using human dermal fibroblasts transfected with a β‐catenin (TOPFLASH) promoter provided evidence that hair follicle induction by eCRT is *via* the Wnt/β‐catenin pathway with the up‐regulation of specific Wnts. Furthermore, a chemical Wnt agonist dose dependently increased Calr transcription and protein synthesis. Further studies revealing the interaction between Calr and Wnt/β‐catenin may be important for potential therapies for deep wound treatment and degenerative skin conditions, and hair loss.

Luis Agellon (Montreal, Canada) presented newly characterized features of Canx deficiency in mice. Adult mice lacking Canx are substantially smaller as compared to their wild‐type littermates (PMID 20400506), which is likely associated with growth arrest occurring between days 15 and 25 after birth, despite the resumption of normal growth rate after day 25. Nutrient absorption in the digestive tract is facilitated by membrane‐bound transporters, and these proteins may require the chaperone function of Canx for proper synthesis. The digestive tract of adult Canx‐deficient mice exhibit changes that appear to be adaptive strategies to ensure nutrient assimilation and survival.

There is a need for new biomarkers that will aid in disease diagnosis, activity and response to therapy. Biomarkers can also be of support in the understanding of disease aetiology. With the expansion of functions ascribed to Calr, there has also been an increase in studies monitoring extracellular Calr and anti‐Calr antibodies in various diseases especially cancers and autoimmune disease. The recent work of Holoshitz and colleagues have demonstrated that the binding of Calr and especially citrullinated Calr (citCalr) to the signal transduction share epitope peptide encoded by a number of major histocompatibility complex class II (HLA‐DRB1) alleles present in a high proportion of rheumatoid arthritis (RA) patients may correlate with erosive bone damage. This hypothesis has been shown to be the case in mouse models of arthritis. Paul Eggleton (Exeter, UK) presented data on the detection of citCalr prior to developing RA and evaluated whether citCalr is a target for autoantibodies in RA cohorts with and without lung disease/bronchiectasis (BR). The detection of citCalr in BR and development of anti‐citCalr in BR patients suggests citCalr antigens are early targets of antigenicity in these patients, prior to the onset of RA.

Nasrin Mesaeli (Doha, Qatar) presented data on a genetically modified mouse model overexpressing Calr under the tunica interna endothelial cell kinase (Tie2) promoter which showed development of metastatic lung adenocarcinoma. Microarray analysis of lung tissue from Tie2‐Calr mice as compared to the wild‐type mice showed a significant increase in a number of long non‐coding RNAs. One of these long non‐coding RNA (LncRNA) known as metastasis‐associated lung adenocarcinoma transcript‐1 (Malat‐1) has been implicated in the development of metastatic lung cancer. Malat‐1 is a novel LncRNA that is localized to nucleus. Furthermore, its 3’ end can be spliced to form tRNA‐like structure called mascRNA that translocate to cytoplasm. Her data illustrate the involvement of this LncRNA in the increased rate of tumour cell migration and altered cell cycle. The exact targets of this LncRNA are not known. Using RNA sequencing analysis of tumour cells after knockdown of Malat‐1, changes were presented in cluster of genes involved in cell proliferation and migration.

## Short presentations by young researchers

During the meeting, a special session was organized for young researchers to short 10 min talks. The session was extremely well received by the participants. Abdullah Treffa (Exeter, UK) discussed mechanisms of Calr binding and release from cancer cells and the implications of her results for immunotherapy; Najla Arshad (New Haven, USA) talked about characterization of tumour‐associated Calr mutants and their effect on antigen presentation by MHC class I; Kristofer Marjon (Stanford, USA) presented data on the secretion of Calr from macrophages to label target cells for phagocytosis; Danilo Medinas (Santiago, Chile) discussed the role of PDIA3 in the pathogenesis of ALS; Sonam Parakh (Sydney, Australia) gave a presentation on ER dysfunction is a common pathological mechanism induced by mutant proteins in ALS; Hector Vega (Edmonton, Canada) presented data on a frameshift mutation in the Calr gene contributing to the pathophysiology of sudden unexplained death; Qian Wang (Edmonton, Canada) presented data on new, a muscle‐specific IRE1α interacting proteins; Wen‐An Wang (Edmonton, Canada) provided evidence how the ER Ca^2+^ homoeostasis dictates the sensitivity of the cellular sterol sensing mechanism 33 and Thomas Balligand (Brussels, Belgium) demonstrated that CRISPR/Cas9‐engineered del52 mutation in the murine Calr gene induces an essential thrombocythemia‐like phenotype and is lethal in the homozygous state.

The current knowledge and future challenges for the involvement of Calr in physiology and pathology are briefly presented in Table 1.

**Table 1:** Current knowledge and future challenges for CALRETICULIN and other ER Components

In Physiology	In Pathology
Structural aspects 1 –Crystallographic data 2 –Interactions with other proteins 3 –Role of post‐translational modifications 4 –Critical mutations Regulatory aspects 1 –Regulation of expression 2 –Role of non‐coding RNAs in transcriptional networks Critical processes 1 –Ca^2+^ signaling 2 –Unfolded Protein Response 3 –Protein degradation 4 –Autophagy 5 –Cell adhesion 6 –Antigen presentation 7 –Wound healing 8 –Nutrient absorption 9 –Sterol sensing	Diseases 1 –Metabolic disorders (obesity, diabetes) 2 –Neurological disorders (Multiple Sclerosis, Amyotrophic Lateral Sclerosis) 3 –Haematological disorders (myeloproliferative diseases, myelofibrosis, essential thrombocytopenia) 4 –Other Cancers (glioblastoma, lung adenocarcinoma) 5 –Renal diseases (diabetic nephropathy, renal fibrosis, allograft rejection) 6 –Chagas Disease Markers for diseases 1 –Detection of extracellular Calr 2 –Detection of anti‐Calr antibodies (in cancer and autoimmunity) Therapeutic prospects 1 –Reversal of ER aggregates

## Conclusions

The 12th International Calreticulin workshop offered an excellent exchange of information on the latest findings in the area of protein structure, protein folding and Ca^2+^ homoeostasis and stress responses with major emphasis on the ER and other intracellular organelles. In addition, as the functions of molecular chaperones expand into the fields of myeloproliferative diseases, neurodegenerative diseases, autoimmune diseases, cancer and metabolism, this workshop provides the optimal venue to bring scientists from diverse disciplines together with a common goal to advance the understanding of the functions of Calr and other ER proteins as relevant therapeutic targets. The 13th International Calreticulin Workshop will be organized in 2019 in the beautiful city of Montreal, Quebec, Canada.

## Conflict of Interest

The authors declare that there is no conflict of interest.
